# Adult *Drosophila* melanogaster as a model for the study of glucose homeostasis

**DOI:** 10.18632/aging.100185

**Published:** 2010-08-05

**Authors:** Aaron T. Haselton, Yih-Woei C. Fridell

**Affiliations:** ^1^ Department of Biology, State University of New York at New Paltz, New Paltz, NY 12561, USA; ^2^ Department of Allied Health Sciences, University of Connecticut, Storrs, CT 06269, USA

**Keywords:** Drosophila insulin-like peptides (DILPs), DILP-producing cells (IPCs), Insulin/Insulin-like growth factor signaling (IIS), hemolymph, adipokinetic hormone (AKH)

## Abstract

Genetic ablation of Drosophila melanogaster insulin-like peptide (DILP) and adipokinetic hormone-producing cells accompanied by cell biological and metabolic measurements have revealed functional conservation in nutrient sensing and the underlying signaling mechanisms between mammal and fruit fly.  Despite significant advances gained in understanding the neuroendocrine responses to nutrient changes during developmental larval stages, we discuss here the need for investigating glucose homeostasis in the post-mitotic adult stage as the result of ablation of DILP producing cells (IPCs).  Our recent studies demonstrate that while both constitutive and adult-specific partial ablation of IPCs renders those flies hyperglycemic and glucose intolerant, flies with adult-specific IPC ablation remain insulin sensitive.  Our results substantiate a role of adult IPCs in modulating aspects of glucose homeostasis and highlight the complexity in DILP action in the adult fly.

## Introduction

The identification and characterization of *Drosophila melanogaster* insulin-like peptides (DILPs) together with accumulating insight into the nutrient sensing and intracellular signaling mechanisms in the brain neurosecretory cells in which DILPs are produced is revealing many important details of the neuroendocrine mechanisms that couple nutrition to metabolic change [1].  These recent discoveries not only advance our understanding of important physiological mechanisms in the fruit fly, they also expand the role of this model organism to include conserved molecular mechanisms of carbohydrate homeostasis in animals.  One concerning limitation common to most studies investigating DILP action to date is that experiments have focused on developmentally immature larval flies, which, in order to facilitate expedited growth and development, exhibit physiologies that may differ significantly from the more metabolically subdued adults. As a major goal of this area of research is to gain insight into common mechanisms that will aid our understanding of mammalian carbohydrate metabolism perturbations, and as many major mammalian metabolic disorders are associated with aging and senescing adults, confirmation of the continuity in homeostatic function from larva to adult is critical in establishing *Drosophila* as an appropriate model for such studies.  In this Research Perspective, we highlight recent work with adult flies that both illustrates the lifelong employment of conserved regulatory mechanisms and demonstrates the value of the adult fly as a relevant system in which to study carbohydrate homeostasis and post-developmental onset of metabolic pathophysiologies in animals.

### Neuroendocrine messengers

**Figure 1.**
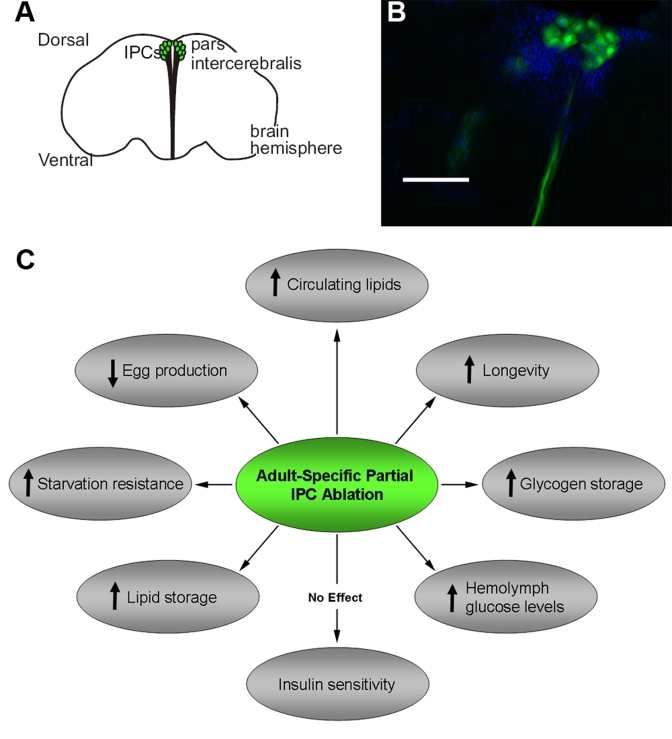
Adult *Drosophila* IPCs modulate metabolism, stress response, fecundity and longevity. A diagram **(A) **and fluorescent micrograph **(B)** of adult DILP producing cells (IPCs) in the *pars intercerebralis *region of the *Drosophila* brain.  The visualization of adult IPCs was achieved via GFP expression in *dilp2*-Gal4/UAS-GFP flies [11]. **C.** A summary of physiological and behavioral effects of adult-specific partial IPC ablation.  Scale bar, 100 μm.

The*Drosophila *genome contains seven *dilp* genes, five of which exhibit significant homology to mammalian insulins [2,3].  *dilp*s are expressed in a variety of tissues including the larval ventral nerve cord, larval salivary glands, larval midgut, ovaries, and the larval and adult brain [2-4].  Most studies investigating the function of these peptide hormones have focused on DILPs 2, 3, and 5, which are all co-expressed in 5-7 pairs of bilaterally symmetrical, clustered median neurosecretory cells in the *pars intercerebralis* (PI) region of the protocerebrum in both larvae and adults (Figure 1A and B) [2-5].  The PI region, in conjunction with the corpus cardiacum/corpus allatum (CC/CA)  tissue  complex of the insect brain  forms  a major component of the neuroendocrine system in the fly analogous to the vertebrate hypothalamus-pituitary axis in both anatomical arrangement and as a master neuroendocrine organ, with embryological evidence suggesting homology [6].  Axonal processes originating from DILP-producing median neurosecretory cells (IPCs) in the PI terminate in neurohemal areas of the aorta and CC tissue-containing ring gland in larvae and presumably the CC portion of the retrocerebral complex in adults, thus providing a route for DILPS to be released directly into the circulatory system [3,4].

### Larval action

Insulin/Insulin-like growth factor signaling (IIS) has been implicated in the regulation of growth, development, metabolism and aging in metazoans [5].  The expression of *dilps2,3, *and *5* are independently regulated in larval IPCs and the expression of *dilps 3* and *5* is regulated by nutrient availability, with starvation reducing the levels of detectable transcripts and leading to peptide accumulation in IPCs and axonal termini [4,7]. Constitutive transgenic ablation of IPCs in *Drosophila* larvae results in growth and developmental impairment, reduced survivorship, and a hyperglycemic phenotype with circulating hemolymph sugar levels 38% above normal [3].  The effect of elevated circulating sugar levels following IPC ablation in immature flies is reminiscent of a mammalian diabetic phenotype and supports the conserved role of DILPs in carbohydrate homeostasis.  Similarly, the effects of life-long constitutive IPC ablation that so radically affects larval physiology manifests in adults as an extension in median and maximum lifespan in both male and female flies, a decrease in egg laying in both mated and virgin female flies, and an increase in oxidative and starvation stress resistance [5]. Additionally, adult female flies that have experienced attenuated insulin signaling throughout development exhibit elevated hemolymph glucose titers (two-fold increase) as well as elevated levels of whole body trehalose, glycogen, and lipids [5].  It is quite clear that reduced insulin signaling experienced throughout development alters normal carbohydrate metabolism and nutrient assimilation, but it has so far been unclear if the effects observed in the adult are due to ongoing processes or to altered development.

The conservation of regulatory endocrine mechanisms controlling circulating glucose levels in *Drosophila* larvae is further evidenced by the presence of cells in the corpus cardiacum that produce and secrete adipokinetic hormone (AKH), which functions in glucose homeostasis by mobilizing stored energy reserves and raising circulating carbohydrate levels [8]. The antagonistic relationship between DILPs and AKH is functionally analogous to that recorded between insulin and glucagon in mammals.  AKH-producing CC cells respond to hypoglycemia with increased intracellular calcium levels, a key step in the signaling cascade that leads to exocytosis of AKH [9].  Hypoglycemic sensing and subsequent exocytosis of AKH in CC cells closely mirrors the function and behavior of mammalian islet α-cells.  Interestingly, flies with ablated AKH-producing CC cells develop and reproduce normally, implying that unlike DILPs, AKH signaling is not essential under normal growth conditions [8].

### Adult action

While a great deal of evidence gathered from the study of *Drosophila* larvae points to the conservation of fundamental endocrine regulatory mechanisms of homeostatic blood sugar levels in insects [8], the larval stage of a holometabolous insect is a unique period dedicated to prodigious nutrient acquisition and rapid growth.  It is therefore possible that *Drosophila* larvae may possess unique metabolic specializations that are not present in adult flies, which have switched over from a growth phase to a largely post-mitotic, reproductive phase.  Evidence for the maintenance of conserved glucose homeostatic mechanisms throughout the *Drosophila* life cycle is accumulating, however, and we report that conditional, adult specific partial IPC ablation yielded a phenotype similar to that seen in larvae experiencing constitutive IPC ablation (Figure 1C) [10].  When subjected to an oral glucose tolerance test, we found that conditionally IPC-ablated adult "knock down" (IPC KD) flies exhibited fasting hyperglycemia and impaired glucose tolerance, yet remained insulin sensitive as measured by peripheral glucose clearance upon insulin injection and serine phosphorylation of a key IIS pathway molecule, Akt [10].  In addition, a moderate increase in median and maximum lifespan, heightened starvation resistance, and reduced early life fecundity are measured as the result of adult-specific, partial IPC ablation.  Thus, these results have confirmed a role of adult IPCs in controlling glucose homeostasis, reproduction, and longevity.

IPC glucose sensing mechanisms also appear to be similar to that of mammalian β-pancreatic cells.  Adult *Drosophila* IPCs expressing the fluorescent Ca^2+^ indicator "camgaroo" (Cg-2) show an increase in fluorescence when exposed to glucose and trehalose, demonstrating that these cells increase their intracellular Ca^2+^ concentration in response to the presence of circulating nutrients [11].  Ca^2+^ influx triggered by the opening of the voltage gated Ca^2+^ channels as the result of closing of the ATP-sensitive K_ATP_ channels is the critical event in insulin release in mammalian β cells [12].  Thus, the mechanism controlling DILPs release from adult IPCs appears conserved.  In supporting this notion, we have reported the detection of transcripts of the sulfonylurea receptor (Sur) functional subunit of K_ATP_ channels in IPCs via *in situ* hybridization [11].

### Conclusion

The tissue level organization of the glucose regulatory system in *Drosophila* is not only analogous to the mammalian islet cell endocrine system [13], but the nutrient sensing and intracellular signaling mechanisms appear to be homologous.  This conserved arrangement of neuroendocrine cells and tissues initially documented in larval flies seemingly survives the dramatic histological rearrangements experienced during insect metamorphosis and continues to monitor and control hemolymph glucose titers in adult flies.  Our recent studies have begun to tease apart key metabolic responses needed to maintain glucose homeostasis in the adult fly, thus establishing the adult fly as an appropriate model for the investigation of adult-specific mechanisms in normal and altered carbohydrate homeostasis.
